# GcsR, a TyrR-Like Enhancer-Binding Protein, Regulates Expression of the Glycine Cleavage System in *Pseudomonas aeruginosa* PAO1

**DOI:** 10.1128/mSphere.00020-16

**Published:** 2016-04-27

**Authors:** Zaara Sarwar, Benjamin R. Lundgren, Michael T. Grassa, Michael X. Wang, Megan Gribble, Jennifer F. Moffat, Christopher T. Nomura

**Affiliations:** a Department of Chemistry, State University of New York—College of Environmental Science and Forestry, Syracuse, New York, USA; b Department of Microbiology and Immunology, State University of New York Upstate Medical University, Syracuse, New York, USA; c Center for Applied Microbiology, State University of New York—College of Environmental Science and Forestry, Syracuse, New York, USA; d Hubei Collaborative Innovation Center for Green Transformation of Bio-Resources, College of Life Sciences, Hubei University, Wuhan, People’s Republic of China; Loyola University Chicago

**Keywords:** Glycine metabolism, *Pseudomonas aeruginosa* PAO1, TyrR, enhancer-binding proteins, transcription factors

## Abstract

Glycine serves as a major source of single carbon units for biochemical reactions within bacterial cells. Utilization of glycine is tightly regulated and revolves around a key group of proteins known as the glycine cleavage system (GCS). Our lab previously identified the transcriptional regulator GcsR (PA2449) as being required for catabolism of glycine in the opportunistic pathogen *Pseudomonas aeruginosa* PAO1. In an effort to clarify and have an overall better understanding of the role of GcsR in glycine metabolism, a combination of transcriptome sequencing and electrophoretic mobility shift assays was used to identify target genes of this transcriptional regulator. It was found that GcsR binds to an 18-bp consensus sequence (TGTAACG-N_4_-CGTTCCG) upstream of the *gcs2* operon, consisting of the *gcvH2*, *gcvP2*, *glyA2*, *sdaA*, and *gcvT2* genes. The proteins encoded by these genes, namely, the GCS (GcvH2-GcvP2-GcvT2), serine hydroxymethyltransferase (GlyA2), and serine dehydratase (SdaA), form a metabolic pathway for the conversion of glycine into pyruvate, which can enter the central metabolism. GcsR activates transcription of the *gcs2* operon in response to glycine. Interestingly, GcsR belongs to a family of transcriptional regulators known as TyrR-like enhancer-binding proteins (EBPs). Until this study, TyrR-like EBPs were only known to function in regulating aromatic amino acid metabolism. GcsR is the founding member of a new class of TyrR-like EBPs that function in the regulation of glycine metabolism. Indeed, homologs of GcsR and its target genes are present in almost all sequenced genomes of the *Pseudomonadales* order, suggesting that this genetic regulatory mechanism is a common theme for pseudomonads.

**IMPORTANCE** Glycine is required for various cellular functions, including cell wall synthesis, protein synthesis, and the biosynthesis of several important metabolites. Regulating levels of glycine metabolism allows *P. aeruginosa* to maintain the metabolic flux of glycine through several pathways, including the metabolism of glycine to produce other amino acids, entry into the trichloroacetic acid cycle, and the production of virulence factors such as hydrogen cyanide. In this study, we characterized GcsR, a transcriptional regulator that activates the expression of genes involved in *P. aeruginosa* PAO1 glycine metabolism. Our work reveals that GcsR is the founding member of a novel class of TyrR-like EBPs that likely regulate glycine metabolism in *Pseudomonadales*.

## Abstract (precis)

Glycine is required for various cellular functions, including cell wall synthesis, protein synthesis, and the biosynthesis of several important metabolites. Regulating levels of glycine metabolism allows *P. aeruginosa* to maintain the metabolic flux of glycine through several pathways, including the metabolism of glycine to produce other amino acids, entry into the trichloroacetic acid cycle, and the production of virulence factors such as hydrogen cyanide. In this study, we characterized GcsR, a transcriptional regulator that activates the expression of genes involved in *P. aeruginosa* PAO1 glycine metabolism. Our work reveals that GcsR is the founding member of a novel class of TyrR-like EBPs that likely regulate glycine metabolism in *Pseudomonadales*.

## INTRODUCTION

Amino acids are a preferred nutrient source for the Gram-negative opportunistic pathogen *Pseudomonas aeruginosa* (1, 2). This bacterium can use almost any amino acid as either a sole carbon or a sole nitrogen source (2). The amino acid glycine is a major source of single carbon units for biochemical reactions in the cell and is necessary for the biosynthesis of several important metabolites such as purines, thymidine, methionine, threonine, and lipids (3–5). Aside from basic metabolism, glycine is also a precursor of hydrogen cyanide (HCN), which *P. aeruginosa* uses as a virulence factor (6).

The metabolism of glycine is dependent on a multienzyme complex known as the glycine cleavage system (GCS), which is composed of three proteins, GcvH, GcvP, and GcvT (7). GcvP is a dehydrogenase that catalyzes the decarboxylation of glycine and transfers the aminomethyl moiety to the lipoyl prosthetic group of GcvH. GcvT is an aminomethyltransferase that mediates the release of ammonia from the intermediate attached to GcvH and the synthesis of 5,10-methylene tetrahydrofolate (5,10-methylene-THF) in the presence of tetrahydrofolate (THF). Overall, the GCS catalyzes the conversion of glycine into carbon dioxide, ammonia, and 5,10-methylene-THF. In addition to the GCS, there are two other proteins that play important roles in glycine metabolism. First, the serine hydroxymethyltransferase (GlyA) transfers a methylene group from 5,10-methylene-THF to glycine, thus forming serine and THF as products. The GlyA reaction is reversible and is necessary for the biosynthesis of glycine from serine (8, 9). The other key protein for glycine metabolism is serine dehydratase (SdaA), which catalyzes the deamination of serine into pyruvate, an intermediate of central metabolism (10, 11). The GCS-GlyA-SdaA pathway provides a route for the utilization of glycine as a carbon and nitrogen source by bacteria.

In the case of *P. aeruginosa* PAO1, two sets of genes coding for GCS proteins are found on the chromosome. There are also three genes coding for GlyA and two serine dehydratase genes in the *P. aeruginosa* PAO1 genome. Among these, one set of glycine metabolism genes, the *gcvH2-gcvP2-glyA2-sdaA-gcvT2* genes, are located together in the chromosome and are collectively known as the *gcs2* cluster. This gene cluster was previously found to be downregulated in the absence of the gene *PA2449* (here *gcsR* [for glycine cleavage system regulator]) (12). Indeed, the *gcsR* gene was also essential for the utilization of glycine as a sole carbon source by *P. aeruginosa* PAO1 (12). The *gcsR* gene encodes a transcriptional regulator belonging to the enhancer-binding protein (EBP) family. EBPs are an interesting family of regulators because they possess domains that enable them to specifically interact with the alternative sigma factor σ^54^ (RpoN) to initiate transcription from their target promoters (13). Upstream of the *gcs2* cluster is a putative RpoN promoter, supporting a model in which GcsR regulates the transcription of *gcs2* genes in response to glycine availability.

In addition to being an EBP, GcsR also shows >40% identity to the TyrR transcriptional regulator of *Escherichia coli*. This further classifies GcsR as a TyrR-like EBP. The members of the TyrR family of regulators are known for their roles in aromatic amino acid biosynthesis and metabolism (14–17). The *P. aeruginosa* PAO1 genome has two genes encoding the TyrR-like EBPs PhhR and GcsR (18). PhhR was previously identified as a regulator of aromatic amino acid metabolism in *P. aeruginosa* PAO1 (16, 19). GcsR shows 44% sequence homology to PhhR (12).

In this study, we show a novel mechanism for the regulation of the *P. aeruginosa* PAO1 *gcs2* cluster by the TyrR-like EBP GcsR. We demonstrate that, unlike other TyrR regulators that respond to aromatic amino acids, GcsR activates the transcription of the *gcs2* cluster in response to glycine. In addition, we show that the five *gcs2* genes are transcribed as an operon and GcsR binds to an 18-bp tandem repeat sequence in the promoter region of the *gcs2* operon to activate transcription. Although the *P. aeruginosa* PAO1 genome contains multiple homologs of each of the *gcvH*, *gcvP*, *gcvT*, *glyA*, and *sdaA* genes, our work indicates that only the *gcs2* operon is essential for the metabolism of glycine as a sole carbon source. GcsR also appears to link glycine metabolism to virulence since the paralytic killing of the nematode *Caenorhabditis elegans* is significantly enhanced in the absence of *gcsR*.

Our work also reveals that the *gcsR* gene is conserved in nearly all of the sequenced genomes of the members of the order *Pseudomonadales* and is found adjacent to genes encoding a GCS. The results presented in this report indicate that GcsR is the prototype of a new family of TyrR-like EBPs that regulate glycine metabolism.

## RESULTS

### GcsR is essential for expression of glycine metabolism genes.

Our previous work had shown that the *gcsR* gene is essential for the metabolism of glycine as a sole carbon source and for pyocyanin production by *P. aeruginosa* PAO1 (12). We also found that more than 300 genes were differentially expressed in the *gcsR* transposon mutant strain PW5126 (12). Curiously, except for the *gcs2* genes, most of the genes that were affected by the disruption in *gcsR* were genes involved in or regulated by quorum signaling. This previous result may be explained by a recent study that showed that many mutants of the *P. aeruginosa* PAO1 transposon mutant library had acquired unrelated mutations that led to altered pyocyanin production and quorum signaling phenotypes (20). It has also been observed in other pathogenic bacteria that transposon mutations can affect the quorum signaling system (21). Therefore, we constructed an in-frame deletion of the *gcsR* gene in *P. aeruginosa* PAO1 (Δ*gcsR* PAO1) to verify if it did indeed affect quorum signaling and pyocyanin production.

Compared to our original transcriptomic study using the *gcsR* transposon mutant strain PW5126, transcriptome sequencing (RNA-Seq) analysis of the Δ*gcsR* PAO1 strain showed that only 20 genes were differentially expressed (Table 1). Interestingly, these did not include any of the quorum-sensing or quorum-regulated genes that had been affected by the *gcsR* transposon mutation. The genes differentially expressed in Δ*gcsR* PAO1 were mostly involved in metabolism. These include the *gcs2* genes that were also downregulated in the *gcsR* transposon mutant. The *gcs2* genes *gcvH2*, *gcvP2*, *glyaA2*, *sdaA*, and *gcvT2* were downregulated 190-, 112-, 28-, 2-, and 3-fold, respectively, in Δ*gcsR* PAO1. Other genes affected in the Δ*gcsR* PAO1 strain include the *narK1*, *narK2*, *narG*, and *narH* genes belonging to a putative operon involved in nitrogen metabolism, which were upregulated ~2.8-fold; an operon containing genes involved in formaldehyde metabolism (*PA3628-adhC*) that was downregulated ~2.5-fold; and an operon encoding small RNAs that regulate iron homeostasis that was downregulated ~2.7-fold. The genes encoding tRNAs for Lys, Met, Ala, and Leu were also downregulated at least 2-fold in the Δ*gcsR* PAO1 strain.

**TABLE 1:** Genes with >2-fold changes in transcript levels in the *P. aeruginosa* Δ*gcsR* PAO1 compared to wild-type *P. aeruginosa* PAO1 grown in PB

Gene ID	Gene name	Mean fold change	Biological function of product
PA0976.1		−2.2	tRNA-Lys
PA1183	*dctA*	−2.08	C_4_-dicarboxylate transport
PA2442	*gcvT2*	−2.7	Glycine metabolism
PA2443	*sdaA*	−2.19	Glycine metabolism
PA2444	*glyA2*	−38	Glycine metabolism
PA2445	*gcvP2*	−111.58	Glycine metabolism
PA2446	*gcvH2*	−188.91	Glycine metabolism
PA3516		2.53	Purine metabolism
PA3628		−2.3	Formaldehyde metabolism
PA3629	*adhC*	−2.46	Formaldehyde metabolism
PA3874	*narH*	3	Nitrogen metabolism
PA3875	*narG*	2.8	Nitrogen metabolism
PA3876	*narK2*	2.85	Nitrogen metabolism
PA3877	*narK1*	2.53	Nitrogen metabolism
PA4153		2.58	Butanediol catabolic process
PA4280.3		−7.5	tRNA-Ala
PA4704.1	*prrF1*	−2.2	Iron homeostasis
PA4704.3	*prrF2*	−3.22	Iron homeostasis
PA4746.1		−2.04	tRNA-Met
PA4937.1		−2.49	tRNA-Leu

**FIG 1:**
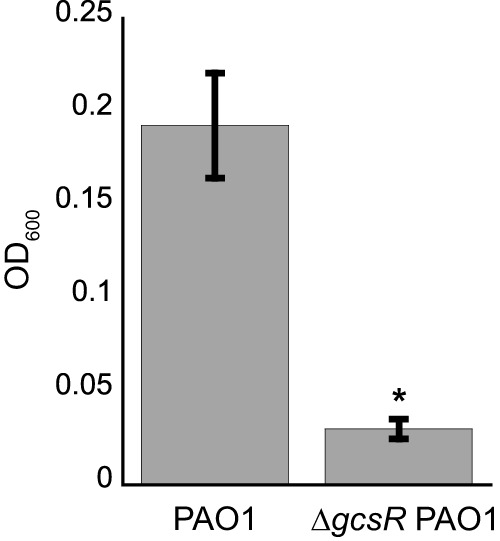
Δ*gcsR* PAO1 is unable to grow in glycine as a sole carbon source. *P. aeruginosa* PAO1 and Δ*gcsR* PAO1 were grown with glycine as the sole carbon source for 48 h at 37°C. Data points represent mean values ± the standard deviations (*n* = 3). Student’s *t* test was performed to identify significant differences (*P* < 0.0001; marked with an asterisk).

The five glycine metabolism genes *gcvH2*, *gcvP2*, *glyA2*, *sdaA*, and *gcvT2* were the only genes that were differentially expressed in both the *gcsR* transposon mutant and the Δ*gcsR* mutant. The transcription levels of these genes were also the most affected in Δ*gcsR* PAO1. Additionally, we found that, like the transposon mutant, the Δ*gcsR* mutant was unable to grow on glycine as a sole carbon source (Fig. 1), providing evidence that the glycine metabolism genes are likely to be an important target of GcsR.

### The *gcs2* cluster genes are cotranscribed in *P. aeruginosa* PAO1.

**FIG 2:**
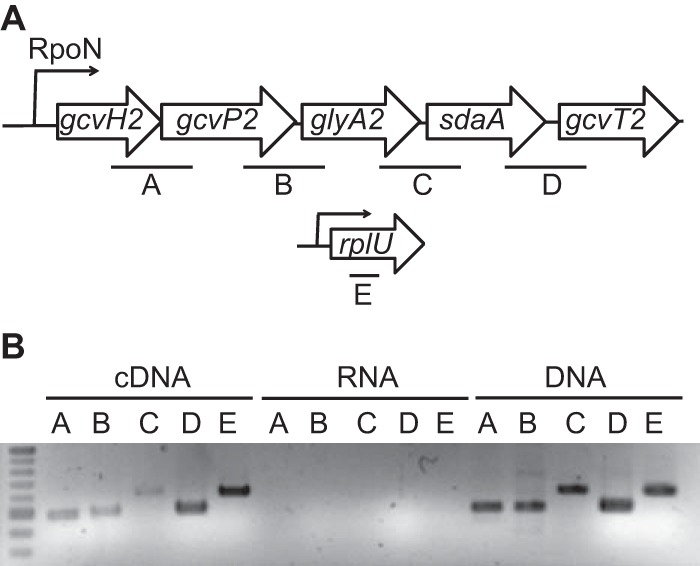
The five *gcs2* genes are transcribed as an operon. (A) At the top is a schematic of the *gcs2* gene cluster. Intergenic regions that were amplified are designated A to D. At the bottom is a schematic of the *rplU* gene, encoding the 50S ribosomal protein (L21), which was was used as a control for the RT-PCR analysis (amplified region designated E). (B) RT-PCR analysis of the *gcs2* gene cluster. The regions designated A to E were amplified by PCR with cDNA, RNA, or genomic DNA obtained from *P. aeruginosa* PAO1 as the template.

The glycine metabolism genes downregulated in the Δ*gcsR* PAO1 strain are arranged together in the *gcs2* gene cluster, with the *gcvH2* gene being the first gene, followed by the *gcvP2*, *glyA2*, *sdaA*, and *gcvT2* genes (Fig. 2A). The *gcvH2*, *gcvP2*, and *gcvT2* gene products together make up the GCS (4), the *glyA2* gene encodes a serine hydroxymethyltransferase that catalyzes the reversible conversion of glycine to serine (9), and *sdaA* encodes a serine dehydratase that catalyzes the deamination of serine to a pyruvate (11). Because of their organization in the genome, their function in glycine metabolism, and the fact that they were all downregulated in the Δ*gcsR* PAO1 strain, we proposed that these genes are cotranscribed and hence regulated by GcsR. In order to test this hypothesis, we determined the operon structure by reverse transcriptase (RT) PCR analysis of RNA isolated from *P. aeruginosa* PAO1 grown for 48 h to an optical density at 600 nm (OD_600_) of 0.25 in minimal medium with glycine as the sole carbon source (Fig. 2B). To check whether adjacent genes were cotranscribed, the cDNA was obtained from the isolated RNA and used as a template for PCR amplification with primers that amplified 500- to 600-bp fragments that spanned intergenic regions from the 3′ end of the upstream gene to the 5′ end of the adjacent downstream gene. PCR products were observed for all four primer sets, indicating that these five genes are transcribed together as a single operon (Fig. 2B).

### Expression of a P*_gcvH2_-lacZ* fusion in *E. coli* is dependent on GcsR and is regulated by glycine availability.

**FIG 3:**
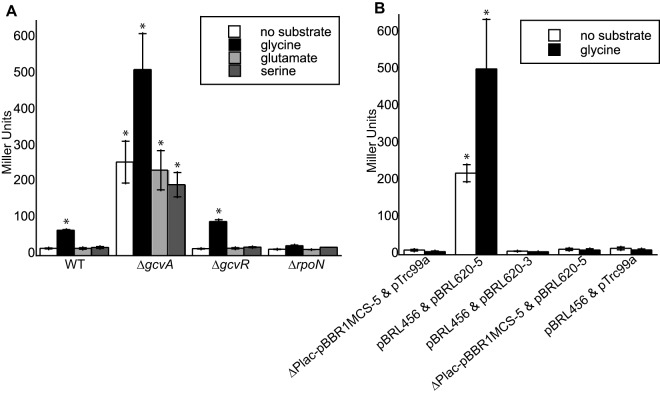
Expression of P_*gcvH2*_::*lacZ* is induced by glycine. (A) Wild-type or Δ*gcvA* or Δ*gcvR* *E. coli* cells harboring the P_*gcvH2*_::*lacZ* reporter construct and the pBRL620-5 plasmid for expression of GcsR were grown in LB to an OD_600_ of ~0.3 and then challenged with 10 mM glycine, 10 mM glutamate, 10 mM serine, or no substrate. (B) *E. coli* Δ*gcvA* mutant cells harboring empty plasmids pTrc99a and ΔP*_lac_*-pBBR1MCS-5, plasmids pBRL456 containing the P_*gcvH2*_::*lacZ* reporter and pBRL620-5 for the expression of GcsR, plasmid pBRL456 and plasmid pBRL620-3 harboring *gcsR* without a promoter, plasmids ΔP*_lac_*-pBBR1MCS-5 and pBRL620-5, or plasmids pBRL456 and pTrc99a were grown in LB to an OD_600_ of ~0.3 and then challenged with 10 mM glycine or no substrate. Data points represent mean values ± the standard deviations (*n* = 3). Analysis of variance was performed by using Dunnett’s *post hoc* test (α value of 0.05) to identify significant differences (*P* < 0.0001; marked with an asterisk).

GcsR was expected to regulate the expression of the *gcs2* operon in response to glycine. To test this hypothesis, the 5′ regulatory region (~500 bp) upstream of *gcvH2* (P_*gcvH2*_) was fused to the β-galactosidase (*lacZ*) open reading frame (ORF) of *E. coli*. The P*_gcvH2_-lacZ* fusion was then cotransformed with a plasmid harboring the *gcsR* gene into nonnative *E. coli*. As shown in Fig. 3A, β-galactosidase (LacZ) levels increased 3-fold with the addition of 10 mM glycine to our recombinant *E. coli* strain. The addition of serine or glutamate had no effect on LacZ levels, suggesting that induction of the P*_gcvH2_-lacZ* fusion was specific to glycine.

Since wild-type *E. coli* can metabolize glycine, the internal glycine concentrations were too low to induce *lacZ* expression, as evidenced by the relatively minor differences in LacZ activity levels in the absence of glycine (20 Miller units [MU]) and in the presence of glycine (70 MU) (Fig. 3A). GcvA is an essential activator of the GCS in *E. coli* (22). Because an *E. coli* Δ*gcvA* mutant is unable to assimilate glycine, the LacZ activity of the P*_gcvH2_-lacZ* fusion was 12-fold higher in this strain than in wild-type *E. coli* (Fig. 3A). In contrast, GcvR is a negative regulator of glycine metabolism in *E. coli* (23), so its absence was not expected to alter the regulation of the P*_gcvH2_-lacZ* fusion by GcsR. Accordingly, in the absence of exogenous glycine, the LacZ level was 259 MU in the Δ*gcvA* mutant compared to 20 and 19 MU in the wild-type and Δ*gcvR* mutant strains, respectively (Fig. 3A). The addition of glycine increased the LacZ level by 2-fold in Δ*gcvA* mutant *E. coli*, which was similar to the fold change observed in wild-type and Δ*gcvR* mutant *E. coli* (Fig. 3A). Lastly, the presence of the *gcsR* gene was required for glycine induction of P*_gcvH2_-lacZ* in *E. coli* (Fig. 3B). Replacement of the plasmid harboring the *gcsR* gene with either an empty plasmid equivalent or a plasmid having the *gcsR* gene in the opposite orientation did not generate significant levels of LacZ activity.

Since GcsR is predicted to be an EBP and since there is a putative RpoN binding site in the P_*gcvH2*_ promoter, we wanted to check whether RpoN is required for transcription from this promoter. We found that an *E. coli* Δ*rpoN* mutant expressing GcsR and harboring the reporter plasmid showed relatively low LacZ levels and no significant difference in LacZ levels in the presence or absence of glycine (17.9 MU in the absence of substrate and 27.7 MU in the presence of glycine) (Fig. 3A). This indicates that RpoN is required for initiation of transcription from the P_*gcvH2*_ promoter.

**FIG 4:**
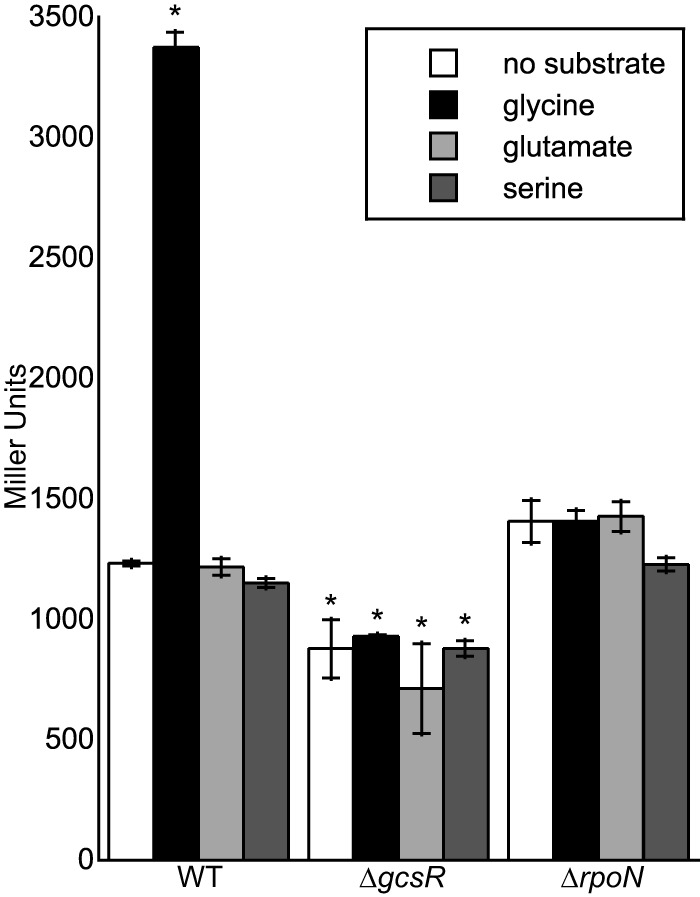
Expression of P_*gcvH2*_::*lacZ* is induced with glycine in *P. aeruginosa* PAO1. *P. aeruginosa* wild-type (WT) PAO1, Δ*gcsR* PAO1, or Δ*rpoN* PAO1 cells harboring the P_*gcvH2*_::*lacZ* reporter construct was grown in M9 minimal medium to an OD_600_ of ~0.3 and then challenged with 10 mM glycine, 10 mM glutamate, 10 mM serine, or no substrate. Data points represent mean values ± the standard deviations (*n* = 3). Analysis of variance was performed by using Dunnett’s *post hoc* test (α value of 0.05) to identify significant differences (*P* < 0.0001; marked with an asterisk).

In order to verify that GcsR regulates the P_*gcvH2*_ promoter *in vivo*, we measured the activity of the P*_gcvH2_-lacZ* fusion in the wild-type PAO1, Δ*gcsR* PAO1, and Δ*rpoN* PAO1 strains (Fig. 4). We found that the LacZ levels in PAO1 increased 3-fold in the presence of exogenous glycine compared to that in cells grown with no substrate or in the presence of serine or glutamate. Additionally, there was no difference in LacZ levels in the presence or absence of glycine in the Δ*gcsR* PAO1 strain or in the Δ*rpoN* PAO1 strain, further indicating that both GcsR and RpoN are required for transcription from the P_*gcvH2*_ promoter.

### GcsR binds to the *gcvH2* promoter region with high specificity and affinity.

**FIG 5:**
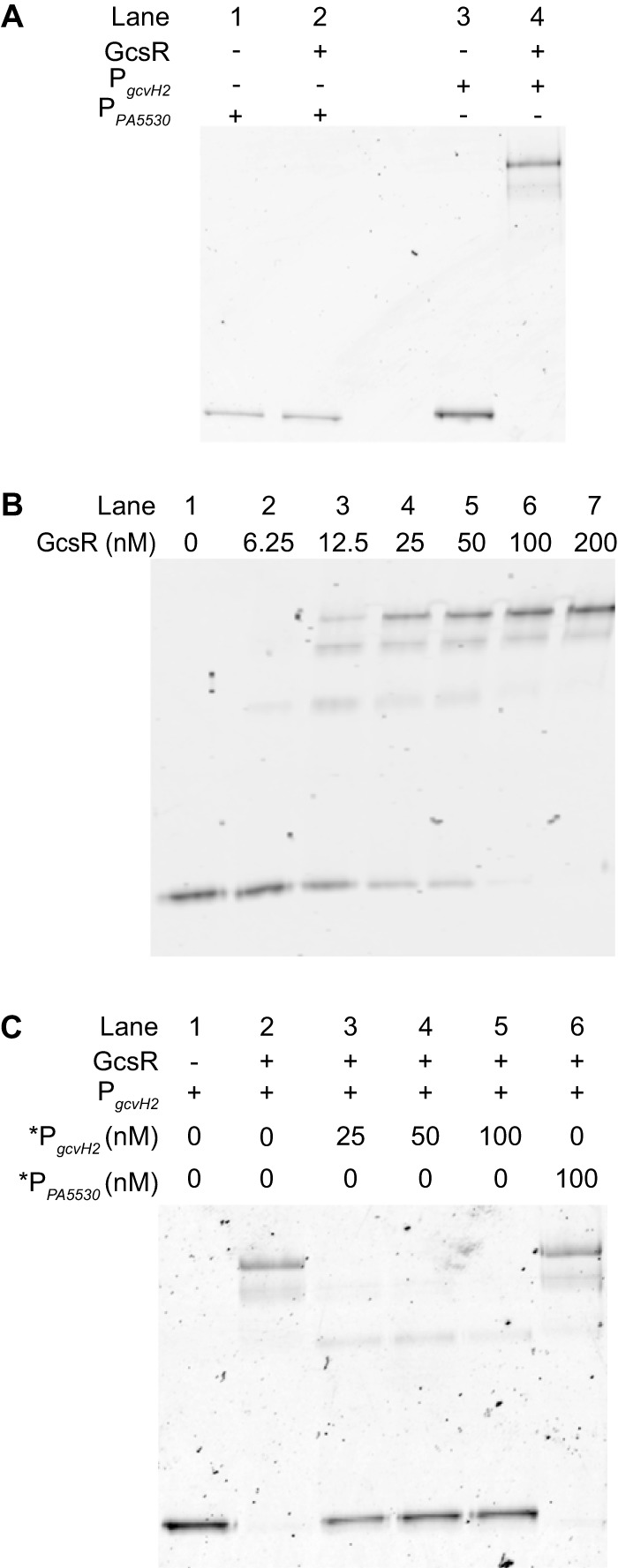
GcsR binds the *gcvH2* promoter region. EMSAs were performed with His_6_-GcsR and 2 nM Cy5-labeled probe DNA unless specified otherwise. (A) P_*PA5530*_ (nonspecific) or P_*gcvH2*_ (specific) was incubated in the absence (lanes 1 and 3, respectively) or presence (lanes 2 and 4, respectively) of 200 nM His_6_-GcsR. (B) His_6_-GcsR (0 to 200 nM) was incubated with P_*gcvH2*_. (C) A 200 nM concentration of His_6_-GcsR was incubated with P_*gcvH2*_ and increasing concentrations of unlabeled specific competitor *P_*gcvH2*_ (lane 3 to 5) or 100 nM unlabeled nonspecific competitor *P_*PA5530*_ (lane 6).

On the basis of homology, the *gcsR* gene encodes a TyrR family EBP (12). Since all of the characterized TyrR family EBPs regulate the metabolism of aromatic amino acids (14, 15), this makes GcsR a unique member of this group. To understand how GcsR regulates glycine metabolism, we first identified its target promoters. The σ^54^ Promoter Database (http://www.sigma54.ca/promoterdata/Web/data.aspx) predicts a putative strong RpoN binding site (score of 92) 79 bp upstream of the predicted *gcvH2* ORF, the first gene of the *gcs2* operon. Furthermore, the *gcs2* operon is located adjacent to the *gcsR* gene in the PAO1 genome (18). This made the *gcvH2* promoter the most likely target for GcsR. To verify this, we used electrophoretic mobility shift assays (EMSAs) to monitor the binding of His_6_-GcsR to a Cy5-labeled DNA probe containing the 200-bp region immediately upstream of the putative RpoN binding site of the *gcvH2* promoter (P_*gcvH2*_). EMSAs showed that His_6_-GcsR was indeed able to bind to P_*gcvH2*_ (Fig. 5A)*.* The affinity of GcsR for this piece of DNA is very high, judging from the shift produced by as little as 6.25 nM His_6_-GcsR, and the intensity of the shift increased with increasing concentrations of His_6_-GcsR (Fig. 5B). We also found that His_6_-GcsR bound to this region with high specificity. To test the specificity of His_6_-GcsR binding, we compared its binding of P_*gcvH2*_ with its binding to the nonspecific probe P_*PA5530*_, which contains a 200-bp region upstream of the RpoN binding site on the *PA5530* promoter (24). Figure 5A shows that while 200 nM His_6_-GcsR completely shifts the P_*gcvH2*_ probe (lane 4), there is no shift in the nonspecific P_PA5530_ probe (lane 2). Also, Fig. 5C shows that addition of the nonlabeled P_*gcvH2*_ probe led to depletion of the shift (lanes 3 to 5), but addition of the nonlabeled P_*PA5530*_ probe did not have any effect on the shift (lane 6). Taken together, these data suggest that the *gcvH2* promoter region is indeed a target for GcsR.

**FIG 6:**
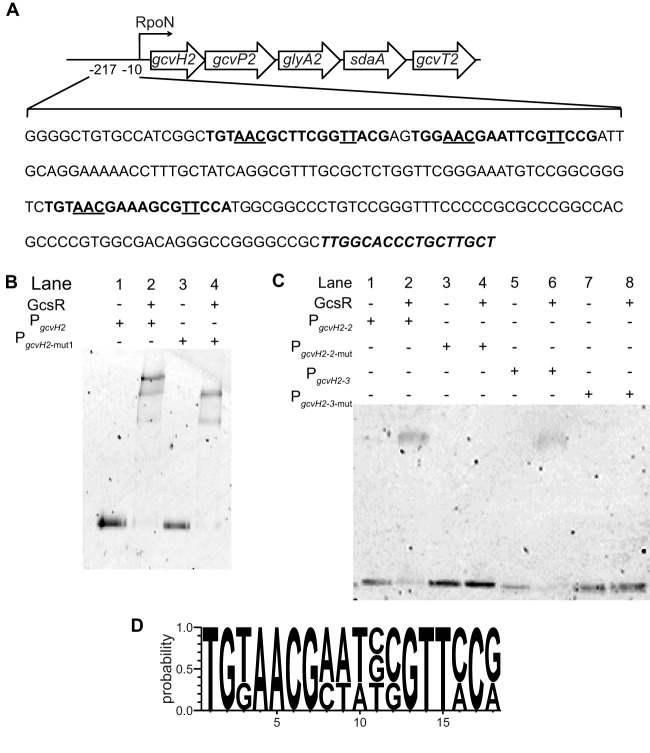
GcsR binds to three 18-bp tandem repeats in the *gcvH2* promoter region. EMSAs were performed with His_6_-GcsR and 2 nM Cy5-labeled probe DNA. (A) Sequence of the 217-bp region upstream of the *gcvH2* gene. The three 18-bp GcsR binding sites are in bold type. The underlined nucleotides in the GcsR binding sites were mutated to G in this study. The RpoN binding site is in bold italic type. (B) A 200 nM concentration of His_6_-GcsR was incubated with P_*gcvH2*_ (lane 2) or P_*gcvH2*-mut1_ (lane 4). (C) P_*gcvH2*-2_, P_*gcvH2*-2-mut_, P_*gcvH2*-3_, and P_*gcvH2*-3-mut_ were incubated in the absence (lanes 1, 3, 5, and 7, respectively) or presence (lanes 2, 3, 6, and 8, respectively) of 200 nM His_6_-GcsR. (D) Consensus sequence of the GcsR binding site derived from the three 18-bp tandem repeats in the P_*gcvH2*_ sequence constructed with WebLogo 3.4.

The EMSAs revealed that the binding of His_6_-GcsR with the P_*gcvH2*_ probe resulted in shifts to three distinct positions (Fig. 5). This suggests that there are at least three binding sites for GcsR in the *gcvH2* promoter region. This is consistent with the binding patterns of known EBPs. EBPs bind as dimers to tandem repeat sequences known as enhancer elements upstream of the RpoN binding site in the target promoter region (13). Typically, three sets of EBP dimers bind to the promoter region. Upon activation, EBPs form a hexameric ring that goes on to interact with RpoN to initiate transcription with the help of ATP hydrolysis. Since we observed three distinct shifts, we expected there to be three GcsR binding sites in the *gcvH2* promoter. Sequence analysis revealed three 18-bp tandem repeat sequences in the 200 bp P_*gcvH2*_ probe (Fig. 6A)*.* In order to investigate the effects of these sequences on GcsR binding, we mutated the first tandem repeat sequence. We found that the binding pattern changed from three shifts with the wild-type sequence to two with the mutated sequence (Fig. 6B). We then inserted mutations into each of the other two tandem repeat sequences separately. We found that GcsR was able to bind each site on its own but the binding was abolished when the individual sites were mutated (Fig. 6C). Taken together, these data indicate that this 18-bp tandem repeat sequence is indeed the binding site for GcsR*.*

Using these three binding sites, we were able to identify the following 18-bp consensus site for GcsR binding: TGTAACG-N_4_-CGTTCCG (Fig. 6D). The binding site is composed of two 7-bp palindromic arms separated by four nucleotides. Upon comparison with the TyrR binding site TGTAAA-N_6_-TTTACA, we find that although the GcsR and TyrR binding sites are very similar, the GcsR binding site has an invariable CG pair at positions 6 and 7 and a CG or GG pair at positions 12 and 13 that is not present at the TyrR binding site.

In addition to the glycine metabolism genes, the RNA-Seq analysis revealed that 15 other genes were differentially expressed in Δ*gcsR* PAO1 (Table 1)*.* However, none of these had a putative RpoN binding site or a putative GcsR binding site and therefore they are probably not direct targets of GcsR.

### GcsR shows TyrR-like phosphatase activity.

**FIG 7:**
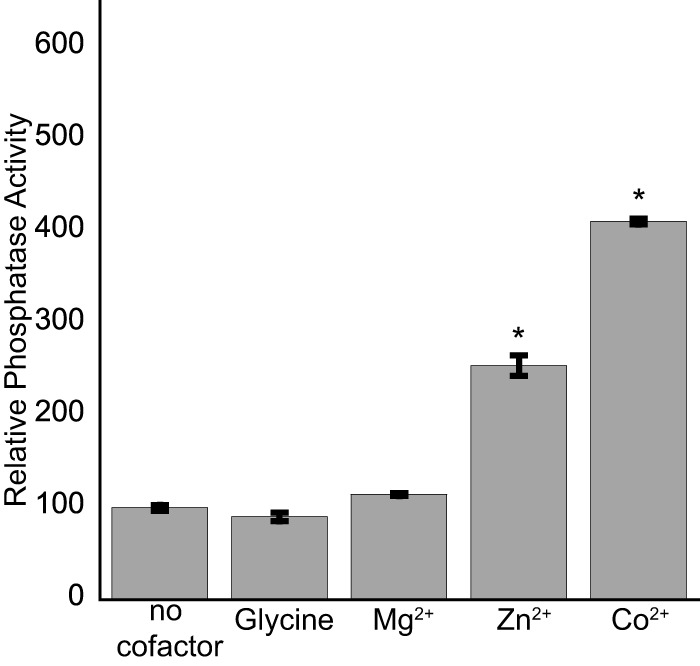
GcsR has divalent-cation-dependent phosphatase activity. A 5 µM sample of His_6_-GcsR was incubated with the substrate *p-*nitrophenylphosphate in the absence of any cofactor or in the presence of 2 mM glycine, 2 mM Mg^2+^, 2 mM Zn^2+^, or 2 mM Co^2+^. Data points represent mean values ± the standard deviations (*n* = 3). Analysis of variance was performed by using Dunnett’s *post hoc* test (α value of 0.05) to identify significant differences (*P* < 0.0001; marked with an asterisk).

The TyrR protein of *E. coli* exhibits phosphatase activity that is dependent on the presence of divalent cations (25). The role of this phosphatase activity *in vivo* has yet to be determined for TyrR. Nonetheless, we decided to determine if GcsR also possesses phosphatase activity *in vitro.* His_6_-GcsR was incubated with or without different divalent cations at 2 mM for 3 h at 37°C. Among the divalent cations tested, Zn^2+^ and Co^2+^ had the most significant effect on GcsR phosphatase activity (Fig. 7). The presence of glycine in the assay mixture did not have any effect on the phosphatase activity of GcsR.

### GcsR is not essential for pyocyanin biosynthesis in *P. aeruginosa* PAO1.

**FIG 8:**
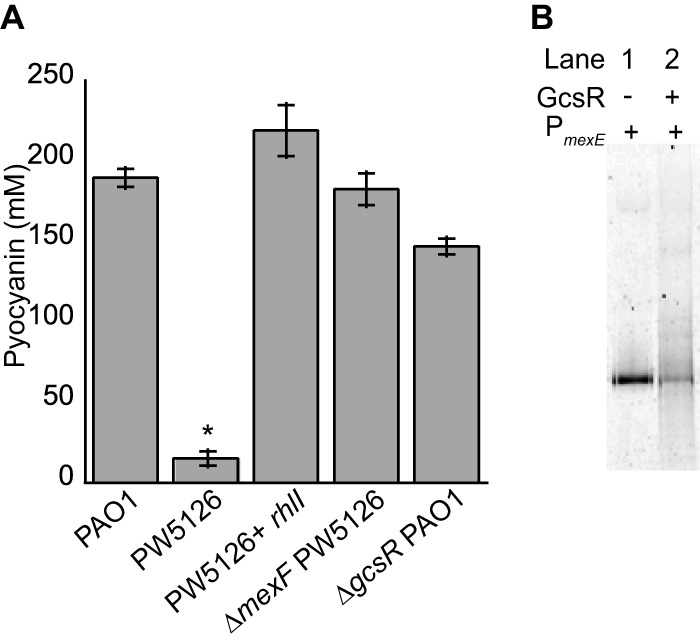
GcsR affects pyocyanin production through the MexEF-OprN efflux pump. (A) Overexpression of *rhlI* or deletion of the *mexF* gene inactivating the MexEF-OprN pump in the *gcsR*::Tn mutant strain PW5126 restores pyocyanin production in PW5126. The Δ*gcsR* PAO1 strain produces pyocyanin levels similar to those of PAO1. Data points represent mean values ± the standard deviations (*n* = 3). Analysis of variance was performed by using Dunnett’s *post hoc* test (α value of 0.05) to identify significant differences (*P* < 0.0001; marked with an asterisk). (B) The P_*mexE*_ probe was incubated in the absence (lane 1) or presence (lane 2) of 200 nM His_6_-GcsR.

Our previous study indicated that GcsR was crucial for the production of pyocyanin in *P. aeruginosa* PAO1 (12). Specifically, a *gcsR* transposon mutant (PW5126) was observed to be deficient in pyocyanin production. Plasmid-derived expression of the *gcsR* gene did restore pyocyanin production in PW5126, suggesting that pyocyanin biosynthesis was dependent on *gcsR*. However, the *gcsR* mutant generated in this study exhibited wild-type levels of pyocyanin production (Fig. 8A), contradicting these previous findings.

A reasonable explanation that might account for the discrepancies in pyocyanin production between the two *gcsR* mutants is the deregulation of quorum sensing-related genes in PW5126. Microarray analysis revealed that numerous quorum sensing genes were significantly downregulated in PW5126 (12). One of these genes was *rhII*, which encodes the *N-*butyryl homoserine lactone (C_4_-HSL) synthase (26, 27). Because C_4_-HSL positively regulates pyocyanin biosynthesis (28), the downregulation of *rhlI* and, consequently, the absence of C_4_-HSL would explain why the PW5126 strain was unable to produce pyocyanin. Consistent with this hypothesis, plasmid-derived expression of *rhlI* did rescue pyocyanin production in PW5126 (Fig. 8A).

In addition to downregulation of *rhlI*, the previous transcriptome analysis indicated that there was >100-fold upregulation of the *mexEF* and *oprN* genes in PW5126. The MexEF-OprN pump has been shown to be responsible for the efflux of biosynthetic precursors of 2-alkyl-4-quinolone, the *Pseudomonas* quinolone signal, which affects the expression of *rhlI* (29–31). Indeed, deletion of the *mexF* gene from PW5126 did restore pyocyanin production (Fig. 8A). This result suggests that upregulation of the *mexEF* and *oprN* genes from PW5126 was the likely cause of its pyocyanin deficiency. Unexpectedly, we found that GcsR did bind with low affinity to the promoter region of *mexEF*-*oprN* (Fig. 8B). This low-affinity binding might explain the previous observation that plasmid-derived expression of *gcsR* rescued pyocyanin biosynthesis in PW5126; i.e., low-affinity binding of GcsR to the P_*mexE*_ promoter region antagonized the transcription of the *mexEF* and *oprN* genes.

### HCN-induced killing of *C. elegans* by Δ*gcsR* PAO1.

One of the metabolic fates of glycine in *P. aeruginosa* is its conversion into HCN through the actions of an HCN synthase (HcnABC). HCN is a virulence factor and causes paralytic killing (HCN-mediated lethal paralysis) of *C. elegans* (32, 33). Since GcsR was necessary for the assimilation of glycine, we wanted to determine if GcsR has any impact on the paralytic-killing capabilities of *P. aeruginosa* PAO1.

**FIG 9:**
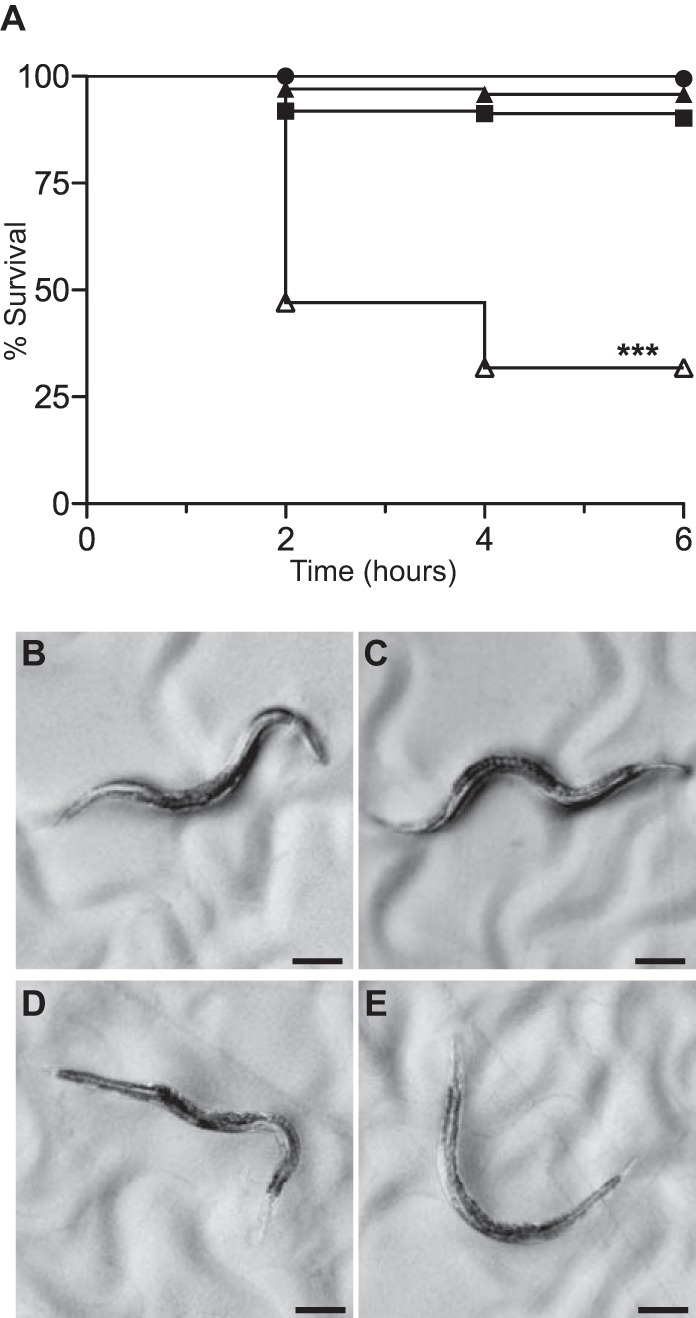
*P. aeruginosa* Δ*gcsR* PAO1 is lethal to *C. elegans* in a paralytic-killing model. (A) Course of *C. elegans* survival in a paralytic-killing assay with *E. coli* OP50 (closed circles; *n* = 195), *P. aeruginosa* lecA::*lux*Δ*lasR* PAO1 (closed squares; *n* = 197), *P. aeruginosa* PAO1 (closed triangles; *n* = 273), and *P. aeruginosa* Δ*gcsR* PAO1 (open triangles; *n* = 351). *C. elegans* survival was significantly reduced when it was exposed to *P. aeruginosa* Δ*gcsR* PAO1. The log rank test was performed to identify significant differences (*P* < 0.0001; marked with asterisks). (B, C) Appearance of *C. elegans* after a 2-h exposure to *P. aeruginosa* PAO1. Scale bars are 100 µm. (D, E) Appearance of *C. elegans* after a 2-h exposure to *P. aeruginosa* Δ*gcsR* PAO1. Scale bars are 100 µm.

After 2 h of exposure to Δ*gcsR* PAO1, ~60% of the worms were dead (Fig. 9A). Figure 9B and C show live, healthy, motile *C. elegans* on a lawn of PAO1 after 2 h of exposure. In contrast, Fig. 9D and E show worms on a lawn of Δ*gcsR* PAO1 after 2 h. The worms in Fig. 9 D and E are dead, as indicated by a lack of pharyngeal pumping. The worm have kinked or curved bodies, as dead *C. elegans* worms commonly do. In some worms, the nose was hypercontracted (data not shown), as previously described (32). *C. elegans* exposed to control strains *P. aeruginosa* lecA::*lux*Δ*lasR* PAO1 and *E. coli* OP50 had motility and appearance similar to those of worms on wild-type *P. aeruginosa* PAO1 lawns (data not shown).

Despite the results of the paralytic-killing assays, RNA-Seq data (see Materials and Methods) showed no significant differences in the expression of the *hcnABC* genes in Δ*gcsR* PAO1 and EMSAs indicated that there was no binding of GcsR to the 5′ upstream regulatory region of *hcnA* (data not shown). This suggests that GcsR does not directly regulate HCN production. Since glycine catabolism is abolished in the GcsR mutant, we hypothesize that more glycine flux into the HCN production pathway is occurring, leading to increased HCN production and lethality.

## DISCUSSION

TyrR family EBPs regulate the expression of genes involved in aromatic amino acid metabolism (14, 15, 19, 34). Many members of this family have been well characterized, including PhhR from *P. aeruginosa* PAO1 (16, 19). Interestingly, in addition to PhhR, *P. aeruginosa* PAO1 has another TyrR homolog (18). The second one is GcsR, and we have found that, unlike other previously characterized TyrR homologs, it regulates the expression of genes required for glycine metabolism.

The primary route of glycine catabolism is catalyzed by the GCS (4). *P. aeruginosa* PAO1 harbors two GCS gene clusters (18). We have found that the *gcs2* gene cluster, comprising the *gcvH2P2T2*, *glyA2*, and *sdaA* genes, forms an operon and is essential for the metabolism of glycine as a sole carbon source. The best-characterized regulation of the expression of a bacterial *gcs* operon is that of *E. coli*, where its expression is regulated by the LysR-type transcriptional activator GcvA (22) and the repressor GcvR (23). Our work has revealed that in *P. aeruginosa* PAO1, a novel TyrR family EBP, GcsR, regulates the expression of this *gcs2* operon and hence the metabolism of glycine. GcsR is the first characterized member of the TyrR family that regulates the metabolism of a nonaromatic amino acid.

GcsR has 41% sequence identity with *E. coli* TyrR. Sequence analysis shows that its domain architecture is identical to that of other TyrR homologs (15, 35): an N-terminal regulatory domain composed of an ACT and PAS domain, a central AAA+ ATPase and RpoN interaction domain, and a C-terminal DNA-binding domain. Like other TyrR family EBPs, GcsR responds to an amino acid effector (glycine). We found that GcsR upregulates the expression of the *gcs2* operon in the presence of glycine. Another piece of evidence indicating that GcsR is a TyrR homolog is that it has divalent-cation-dependent phosphatase activity like *E. coli* TyrR (25). How GcsR binds to metals is unclear since its sequence does not have any recognizable metal-binding motifs such as stretches of multiple cysteine or histidine residues. Although the exact purpose of the phosphatase activity of TyrR or GcsR is not known, one possibility is that GcsR regulates expression from its target promoter by dephosphorylating other phosphorylated proteins. Further work is needed to better characterize the phosphatase activity of GcsR.

**FIG 10:**
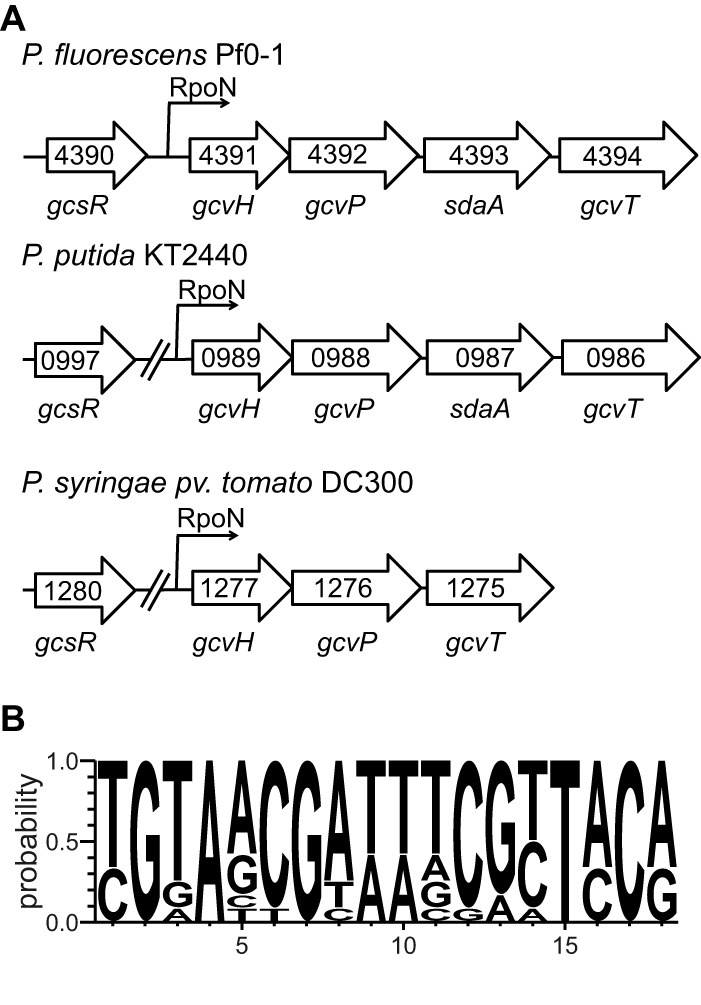
The *P. fluorescens* Pf0-1, *P. putida* KT2440, and *P. syringae* pv. tomato DC3000 genomes harbor *gcsR* orthologs. (A) Organization of the *gcsR* gene and the *gcs* gene cluster in *P. fluorescens* Pf0-1, *P. putida* KT2440, and *P. syringae* pv. tomato DC3000. (B) Consensus sequence of putative binding sites of GcsR orthologs from *P. fluorescens* Pf0-1, *P. putida* KT2440, and *P. syringae* pv. tomato DC3000 constructed with WebLogo 3.4.

*P. aeruginosa* PAO1 is not the only pseudomonad with a genome that harbors a *gcsR* gene. We found that there are GcsR orthologs in a number of other sequenced genomes (Table 2). All of the genomes that harbor a GcsR ortholog belong to the order *Pseudomonadales*. Additionally, all of these GcsR orthologs are located adjacent to a glycine metabolism gene cluster (18) (Fig. 10A). This likely indicates a conserved mechanism for regulating glycine metabolism in members of the order *Pseudomonadales*.

**TABLE 2:** Members of the order *Pseudomonadales* harboring GcsR orthologs

Bacterial strain	GcsR ortholog ID^a^
*Azotobacter chroococcum* NCIMB 8003	AChR_RS10790
*Azotobacter vinelandii* Avop	Avin_25940
*Pseudomonas alcaligenes* OT 69	L682_31660
*Pseudomonas amygdali* 2250	IC51_RS0115310
*Pseudomonas brassicacearum* DF41	CD58_RS22465
*Pseudomonas chlororaphis* O6	PchlO6_4751
*Pseudomonas cremoricolorata* ND07	LK03_RS10195
*Pseudomonas denitrificans* 106_PDEN	ADM39_RS28200
*Pseudomonas entomophila* L48	PSEEN4432
*Pseudomonas fluorescens* Pf0-1	PFL01_4390
*Pseudomonas fragi* A22	O5G_RS0105995
*Pseudomonas fuscovaginae* CB98818	Y53_RS0126010
*Pseudomonas mandelii* JR	OU5_RS24435
*Pseudomonas mendocina* ymp	Pmen_1348
*Pseudomonas monteilii* SB3078	X969_03350
*Pseudomonas mosselii* SJ10	O165_RS17570
*Pseudomonas oleovorans* MGY01	GL31_RS19330
*Pseudomonas oryzihabitans* RIT370	UM91_RS16980
*Pseudomonas parafulva* CRS01-1	NJ69_RS01535
*Pseudomonas plecoglossicida* NyZ12	RK21_RS19015
*Pseudomonas protegens* Pf-5	PFL_4639
*Pseudomonas pseudoalcaligenes* ad 6	AU05_RS20595
*Pseudomonas psychrophila* DSM 17535	TU76_RS13130
*Pseudomonas putida* KT2440	PP_0997
*Pseudomonas resinovorans* NBRC 106553	PCA10_RS15640
*Pseudomonas savastanoi* BO76	PsgB076_20487
*Pseudomonas simiae* WCS417	PS417_RS06035
*Pseudomonas syringae* pv. tomato DC3000	PSPTO_1280
*Pseudomonas taiwanensis* DSM 21245	H620_RS0100750
*Pseudomonas thermotolerans* DSM 14292	H165_RS0110300
*Pseudomonas tolaasii* PMS117	PTOL117_RS0124240
*Pseudomonas umsongensis* 20MFCvi1.1	D470_RS0108450
*Pseudomonas veronii* 1YB2	Y055_RS30055
*Pseudomonas viridiflava* CC1582	N029_RS0112150

a GcsR ortholog sequences are >80% identical to the *P. aeruginosa* PAO1 GcsR protein.

The binding site of GcsR is an 18-bp tandem repeat sequence with two palindromic arms that is very similar to that of TyrR binding sites (Fig. 6D). We analyzed the genome sequences of *P. putida* KT2440, *P. fluorescens* Pf0-1, and *P. syringae* pv. tomato DC300 and identified binding sites for GcsR in the 5′ regulatory regions of the *gcs* gene clusters in these genomes (Fig. 10B). The σ^54^ Promoter Database (http://www.sigma54.ca/promoterdata/Web/data.aspx) indicates that there are putative RpoN binding sites upstream of the *gcs* cluster in all three strains. Additionally, RpoN has been shown to be required for glycine metabolism in *P. putida* KT2440 (36). Taken together, these data suggest that the regulation of glycine metabolism by GcsR and RpoN is likely a common mechanism among members of the order *Pseudomonadales*.

The *P. aeruginosa* genome harbors two homologs of the *gcvHPT* genes, three homologs of the *glyA* gene, and two homologs of the l-serine dehydratase genes, namely, *sdaA* and *sdaB.* However, this does not translate to redundancy in the function of the glycine metabolism genes. The *glyA1* and *sdaB* genes have been shown to function in the glycine betaine catabolism pathway, and their expression is regulated by the AraC-like transcriptional activator GbdR (37, 38). Our work indicated that only the *gcs2* operon, *gcvH2-gcvP2-glyA2-sdaA-gcvT2*, is required for the metabolism of glycine as the sole carbon source. We also found that the growth defect displayed by Δ*gcsR* PAO1 with glycine as the sole nitrogen source was much less pronounced than the growth defect with glycine as the sole carbon source. This suggests that GcsR and, hence, the GCS2 proteins are not essential for the metabolism of glycine as a nitrogen source. Both the GCS and GlyA have been shown to be important for the metabolism of glycine as a nitrogen source in *E. coli* (39). However, since GcvH2P2T2 or GlyA2 does not appear to be essential for the metabolism of glycine as a nitrogen source by *P. aeruginosa* PAO1, it is likely that the other *gcv* genes and perhaps the *glyA3* gene are involved in that process. This is an excellent example of the diverse metabolic capacity of *P. aeruginosa* PAO1.

Glycine also acts as the precursor for the production of HCN gas by an HCN synthase in *P. aeruginosa* (6). *P. aeruginosa* is one of the few bacteria that produce HCN (40). HCN has been detected in patients with *P. aeruginosa* infection and is thought to contribute to the pathogenesis of *P. aeruginosa* in cystic fibrosis patients (41). Although how HCN production by *P. aeruginosa* contributes to its virulence in cystic fibrosis lungs is not yet clear, it has been shown that *P. aeruginosa* uses HCN for paralytic killing of the nematode *C. elegans* (33). We found that the level of *C. elegans* paralytic killing by Δ*gcsR* PAO1 was much greater than that by the parent *P. aeruginosa* PAO1 strain. Since the GCS is not functioning in the Δ*gcsR* PAO1 mutant, more glycine is available for conversion to HCN, which is likely responsible for the increased lethality of the Δ*gcsR* PAO1 strain.

We had previously found that, in addition to regulating glycine metabolism, GcsR also plays a role in pyocyanin production (12). Pyocyanin is one of several redox-active phenazines produced by *P. aeruginosa* that are used as virulence factors (42–47). Pyocyanin causes virulence by affecting gene expression, electron transport, cellular respiration, and innate immune mechanisms (42, 46, 47). We had previously found that pyocyanin production is reduced in the *gcsR* transposon mutant strain PW5126 (12). In this study, we found that this decrease in production of pyocyanin in PW5126 is due not to the direct regulation by GcsR of biosynthetic genes but to overexpression of the MexEF-OprN efflux pump. However, we found that this was not reproducible in the Δ*gcsR* PAO1 strain. When we tested the pyocyanin production of this strain, we found that it was not significantly reduced. Furthermore, in contrast to PW5126, RNA-Seq analysis of Δ*gcsR* PAO1 showed no significant change in *mexEF-oprN* expression. The parent strain of the *gcsR* transposon mutant strain PW5126 is the PAO1 strain from Colin Manoil’s lab at the University of Washington (48). Our Δ*gcsR* PAO1 strain is a derivative of a different PAO1 strain from the Dieter Haas lab at the University of Lausanne in Switzerland. It has been shown that in many PAO1 strains originating from different laboratories, *mexEF-oprN* is quiescent and uninducible because of mutations in several regulatory genes (49, 50). Recent studies have also shown differences in the genome sequences of different PAO1 strains originating from different laboratories (51). Additionally, unexpected mutations affecting the expression of *mexEF-oprN* have also been shown to alter the pyocyanin production of several PAO1 strains and a number of mutants from the PAO1 transposon library (20). Thus, one explanation for this discrepancy could be that the PW5126 strain had an inducible *mexEF-oprN* operon, whereas Δ*gcsR* PAO1 did not. The other possibility is that an adventitious mutation that allows the overexpression of *mexEF-oprN* could have occurred in PW5126 because of the transposon mutation. Further studies must be conducted with known *mexEF-oprN*-inducible strains to fully understand the effect of GcsR on *mexEF-oprN.*

Our RNA-Seq analysis of the Δ*gcsR* PAO1 strain revealed that 20 genes were differentially regulated (Table 1). In addition to the five *gcs2* operon genes, these genes include genes for several tRNAs, an operon encoding genes for formaldehyde metabolism, and another operon involved in nitrogen metabolism. However, none of the latter 15 genes are likely to be direct targets of GcsR. There are two lines of evidence to suggest this. First, we could not identify a putative GcsR binding site in the 5′ regulatory region of any of these genes. Second, 14 out of these 15 genes do not have an RpoN binding site in their promoter region. The only gene with an RpoN binding site in its promoter region is *dctA*, which has been previously shown to be regulated by the DctD EBP (52). This, along with the fact that the *dctA* promoter region lacks a GcsR binding site, indicates that *dctA* is likely not a direct target of GcsR. All of these genes function in pathways that directly or indirectly depend on products from the glycine metabolism pathway. This could possibly explain why disruption of glycine metabolism likely causes changes in their expression patterns.

In conclusion, our data suggest that GcsR is the first characterized member of a novel family of TyrR-like EBPs that regulate genes required for glycine metabolism. Unlike *E. coli*, which uses a LysR-type regulator to regulate glycine metabolism, GcsR also presents a novel mechanism of regulation of glycine metabolism involving the alternative σ factor RpoN. Future work will determine how GcsR interacts with glycine to regulate the expression of its target genes.

## MATERIALS AND METHODS

### Bacterial strains, plasmids, and growth conditions.

The bacterial strains, plasmids, and oligonucleotides used in this study are listed in Table 3. Bacteria were grown in Difco Lennox broth (LB), peptone broth (PB) (12), modified M9 minimal medium supplemented with 5 µM FeSO_4_ (12), 2×YT medium (53), or brain heart infusion (BHI) broth (RPI). Solid bacteriological medium was prepared with the addition of BD agar at 15 g liter^−1^, unless otherwise specified. Liquid cultures were grown at 37°C with shaking at 200 rpm, and cultures on solid plates were grown by incubation at 37°C. When required, the antibiotics carbenicillin (Cb; 100 µg·ml^−1^ for *E. coli* or 200 µg·ml^−1^ for *P. aeruginosa*), kanamycin (Km; 50 µg·ml^−1^ for *E. coli*) or gentamicin (Gm; 10 µg·ml^−1^ for *E. coli* or 30 µg·ml^−1^ for *P. aeruginosa*) were added to culture medium to maintain plasmids.

**TABLE 3:** Bacterial strains, plasmids, and oligonucleotides used in this study

Strain, plasmid, or oligonucleotide	Features	Source
*P. aeruginosa*		
PAO1	Wild type	61
Δ*gcsR* PAO1	Δ*gcsR* derivative of PAO1	This study
PW5126	*gcsR*-E03::IS*phoA*/*hah*	48
ΔmexF PW5126	Δ*mexF* derivative of PW5126	This study
lecA::luxΔlasR PAO1	Δ*lasR* derivative of *lecA*::*lux PAO1*	62
*E. coli*		
JW2779-5	Δ*gcvA736*::Km^r^	63
JW2464-1	Δ*gcvR727*::Km^r^	63
BL21	*fhuA2* [*lon*] *ompT gal* (λ DE3) [*dcm*] Δ*hsdS*	EMD Millipore
Top10	F^−^ *mcrA* Δ(*mrr*-*hsdRMS*-*mcrBC*) φ80*lacZ*ΔM15Δ*lacX74 nupG recA1araD139* Δ(*ara*-*leu*)*7697 galE15 galK16 rpsL* (Str^r^) *endA1* λ^−^	Invitrogen
OP50	Strain used for *C. elegans* growth	59
Plasmids		
pBBR1MCS-5	Broad-host-range plasmid; Gm^r^	64
ΔP*_lac_*-pBBR1MCS-5	pBBR1MCS-5 minus *lac* promoter; Gm^r^	12
pCR-Blunt	Cloning plasmid; Km^r^	Invitrogen
pET28b	Expression plasmid; Km^r^	EMD Millipore
pTrc99a	Expression plasmid; Cb^r^	Pharmacia
pDONR221	Cloning plasmid; Km^r^	Invitrogen
pEX18ApGW	Plasmid for gene deletions in *P. aeruginosa*; Cb^r^	54
pBRL408	*gcsR* in pCR-Blunt; Km^r^	This study
pBRL417	*gcsR* in pET28b; Km^r^	12
pBRL620-3	*gcsR* cloned in backwards in pTrc99a; Cb^r^	This study
pBRL620-5	*gcsR* cloned in forwards in pTrc99a; Cb^r^	This study
pBRL456	P*_gcvH2_*-*lacZ* fusion in ΔP*_lac_*-pBBR1MCS-5; Gm^r^	12
pBRL518	*mexF*::Gm^r^ in pDONR221; Gm^r^ Km^r^	This study
pBRL521	*mexF*::Gm^r^ in pEX18ApGW; Cb^r^ Gm^r^	This study
pBRL527	*gcsR*::Gm^r^ in pDONR221; Gm^r^ Km^r^	This study
pBRL528	*gcsR*::Gm^r^ in pEX18ApGW; Cb^r^ Gm^r^	This study
pMTG01	*rhlI* in pCR-Blunt; Km^r^	This study
pMTG02	*rhlI* in pBBR1MCS-5; Gm^r^	This study
pZS406	P_*gcvH2*_ probe in pJET1.2; Cb^r^	12
pZS416	P_*PA5530*_ probe in pJET1.2; Cb^r^	This study
pZS420	P_*gcvH2*-*mut1*_ probe in pJET1.2; Cb^r^	This study
Oligonucleotides		
BL439.f	GCATCTAGAAGAAGGAGACATATGATCGAATTGCTCTCTGAATCG	*rhlI* gene with XbaI at start
BL439.r	GCAGAGCTCTTCACACCGCCATCGACAGC	*rhII* gene with SacI at stop
BL452.f	TACAAAAAAGCAGGCTGCATCCACGTCTCCTTCATC	*gcsR* UpF-GWL
BL452.r	TCAGAGCGCTTTTGAAGCTAATTCGCAACGCATCGAGCTGCAAG	*gcsR* UpR-Gm
BL453.f	AGGAACTTCAAGATCCCCAATTCGCACTTCATGCAGCAGGCCTG	gcsR DnF-Gm
BL453.r	TACAAGAAAGCTGGGTCTGACGTAGAGCTTCTCCAG	*gcsR* DnR-GWR
BL454.f	TACAAAAAAGCAGGCTGAATTTCTCCCAATTCTTCATCC	*mexF* UpF-GWL primer
BL454.r	TCAGAGCGCTTTTGAAGCTAATTCGGAAGGTGATGGTCAGGGTC	*mexF* UpR-Gm
BL455.f	AGGAACTTCAAGATCCCCAATTCGGCAAGAACGCGATCCTGATC	*mexF* DnF-Gm
BL455.r	TACAAGAAAGCTGGGTCATGCATGCACCTCTGGCAG	*mexF* DnR-GWR
BL456.f	GGCTCGAGTTTTTCAGCAAGAT	pJET1.2
BL456.r	GAATATTGTAGGAGATCTTCTAGAAAG	pJET1.2
JRH05.f	GGCTCGAGTTTTTCAGCAAGAT	5′ Cy5-labeled pJET1.2
JRH05.r	GAATATTGTAGGAGATCTTCTAGAAAG	5′ Cy5-labeled pJET1.2
ZS420F	CGTTCCACTCGTCCCCGAAGCCCCACAGCCGATGGCACATCTTGCTG	P_*gcvH2*-mut1_
ZS420R	CAGCAAGATGTGCCATCGGCTGTGGGGCTTCGGGGACGAGTGGAACG	P_*gcvH2*-mut1_
ZS426F	GGCTCGAGTTTTTCAGCAAGATGAGTGGAACGAATTCGTTCCGATTGCAGGAAAAACCTT	P_*gcvH2-2*_
ZS426R	TTGTAGGAGATCTTCTAGAAAGAAGGTTTTTCCTGCAATCGGAACGAATTCGTTCCACTC	P_*gcvH2*-*2*_
ZS422F	GGCTCGAGTTTTTCAGCAAGATGAGTGGGGCGAATTCGGGCCGATTGCAGGAAAAACCTT	P_*gcvH2*-*2*-mut_
ZS422R	TTGTAGGAGATCTTCTAGAAAGAAGGTTTTTCCTGCAATCGGCCCGAATTCGCCCCACTC	P_*gcvH2*-*2*-mut_
ZS431F	GGCTCGAGTTTTTCAGCAAGATGGCGGGTCTGTAACGAAAGCGTTCCATGGCGGCCCTG	P_*gcvH2*-*3*_
ZS431R	TTGTAGGAGATCTTCTAGAAAGCAGGGCCGCCATGGAACGCTTTCGTTACAGACCCGCC	P_*gcvH2*-*3*_
ZS425F	GGCTCGAGTTTTTCAGCAAGATGGCGGGTCTGTGGCGAAAGCGCCCCATGGCGGCCCTG	P_*gcvH2*-*3*-mut_
ZS425R	TTGTAGGAGATCTTCTAGAAAGCAGGGCCGCCATGGGGCGCTTTCGCCACAGACCCGCC	P_*gcvH2*-*3*-mut_
ZS416F	CACTGAGAGCCAAGCGATC	P_*PA5530*_
ZS416R	CGCGGGAAAGCCGCGTGGGGAC	P_*PA5530*_
ZS448F	ACGCCGCCGGCAGTGAAGTG	*gcvH2*-*gcvP2* RT probe
ZS448R	TTGCGCGCGGCGATCGCC	*gcvH2*-*gcvP2* RT probe
ZS449F	CCGCCGCCGAACTGCTCG	*gcvP2*-*glyA2* RT probe
ZS449R	GGATAGCCCTCGGCATACTTG	*gcvP2*-*glyA2* RT probe
ZS454F	CAGGGGCTGACCGGCAAG	*glyA2*-*sdaA* RT probe
ZS454R	GCTGGAACGGATGGCATCG	*glyA2*-*sdaA* RT probe
ZS451F	GTCCAGGTGCCCTGCATCG	*sdaA*-*gcvT2* RT probe
ZS451R	GCCACATCGGCGCCCACC	*sdaA*-*gcvT2* RT probe
ZS452F	CAAGGTCGAGAAACTCGACGTCG	*rplU* RT probe
ZS452R	GACGCTTCATGTGGTGCTTACGAC	*rplU* RT probe

### Construction of *P. aeruginosa* PAO1 deletion mutant strains.

The Δ*gcsR* PAO1 and Δ*mexF* PW5126 strains were constructed by previously described methods (24, 54) Briefly, the *gcsR*::Gm and *mexF*::Gm cassettes were cloned into pEX18ApGW to give plasmids pBRL528 and pBRL521, respectively. The *gcsR* gene was deleted from PAO1 with the pBRL528 plasmid, and the *mexF* gene was deleted from PW5126 with the pBRL521 plasmid. The Gm markers were removed with the pFLP2 plasmid. The Δ*gcsR* and Δ*mexF* mutations were verified by PCR.

### Preparation of RNA.

RNA of *P. aeruginosa* PAO1 and Δ*gcsR* PAO1 were isolated and purified as described previously (12). To isolate RNA for RNA-Seq, each strain was grown in quadruplicate in 50 ml of PB in 500-ml baffled shake flasks at 37°C and 200 rpm to an OD_600_ of ~0.5. To isolate RNA for RT analysis of operon organization, each strain was grown in quadruplicate in 50 ml of M9 minimal medium supplemented with 20 mM glycine as the sole carbon source in 500-ml baffled shake flasks at 37°C and 200 rpm to an OD_600_ of ~0.25. Cultures were immediately stabilized by adding 1 ml of RNAprotect Bacteria reagent (Qiagen) to 0.5 ml of culture. Cells were then lysed with lysozyme and proteinase K as described in the manufacturer’s protocol. The total RNA was subsequently purified from the lysed cells with the RNeasy minikit (Qiagen) by using an on-column DNase digestion step. PCR and a Bioanalyzer were used to check the RNA for DNA contamination and quality.

### RNA-Seq.

The purified total RNA was sent to the Molecular Analysis Core at SUNY Upstate Medical University for mRNA isolation, cDNA library preparation, and RNA sequencing with the Illumina NextSeq 500 system. CLC Genomics workbench 8.5 (Qiagen) was used to map the sequencing reads to the *P. aeruginosa* PAO1 genome and to obtain differential gene expression data (a ≥2-fold change in the number of reads per kilobase of transcript per million mapped reads and a Bonferroni-corrected *P* value of ≤0.05) (55, 56).

### Analysis of operon organization of glycine metabolism genes.

In order to map the operon organization of the *gcs2* locus, RT analysis of isolated RNA was used. An RT reaction with 500 ng of each RNA sample was set up with the iScript cDNA synthesis kit (Bio-Rad) to obtain cDNA. Primers ZS448F/ZS448R, ZS449F/ZS449R, ZS454F/ZS454R, and ZS451F/ZS451R were designed to amplify 500- to 600-bp regions spanning from the 3′ end of *gcvH2* to the 5′ end of *gcvP2*, from the 3′ end of *gcvp2* to the 5′ end of *glyA2*, from the 3′ end of *glyA2* to the 5′ end of *sdaA*, and from the 3′ end of *sdaA* to the 5′ end of *gcvT2*, respectively. Primers ZS452F/ZS452R were designed to amplify the 50S ribosomal protein L21 gene *rplU* as a positive control (57). PCR analyses of the cDNA samples were subsequently performed with these primers. PCR analyses of the RNA samples and genomic DNA samples were performed as negative and positive controls, respectively. The PCR products obtained were analyzed by agarose gel electrophoresis.

### LacZ reporter assays.

Recombinant *E. coli* strains were grown in M9 minimal medium supplemented with glycine, glutamine, or serine at a 20 mM concentration. Each culture was grown in triplicate. Cells were grown to an OD_600_ of 0.3. β-Galactosidase (LacZ) activity was measured with the Miller assay as described previously (12).

### Heterologous expression and purification of GcsR.

GcsR was expressed and purified as an N-terminally six-histidine-tagged fusion protein (His_6_-GcsR) from plasmid pBRL417 harboring the *gcsR* gene using *E. coli* BL21(DE3) as described previously (53). Cells were grown in 2×YT medium to an OD_600_ of ~0.6 at 37°C with shaking at 200 rpm. Protein expression was induced by the addition of 0.1 mM isopropyl-β-d-thiogalactopyranoside, and cell cultures were incubated for 12 h at 16°C with shaking at 200 rpm. Cells were harvested, resuspended in lysis buffer (100 mM sodium phosphate [pH 8.0], 300 mM NaCl, 10% glycerol, 1 mg/ml lysozyme, 5 U/ml DNase I, 1 µg/ml pepstatin, 1 µg/ml leupeptin), and lysed by three 30-s sonication bursts. The lysate was centrifuged at 9,000 × *g* to remove insoluble material, and the His_6_-GcsR was then purified with Ni-nitrilotriacetic acid Superflow resin (Qiagen). The protein was eluted off the resin by a step elution method with elution buffer (100 mM Tris [pH 8.0], 300 mM NaCl) containing 20, 100, and 250 mM imidazole. Purified protein was concentrated with Amicon Ultra centrifugal filter units (Millipore). Protein expression and purification were monitored visually by sodium dodecyl sulfate-polyacrylamide gel electrophoresis (PAGE). The concentration of purified protein was determined with the Bradford assay.

### EMSAs.

DNA fragments containing the promoter region of the *gcvH2* gene were amplified by PCR and cloned into the pJET1.2 vector. The mutated promoter regions were obtained with a quick-change site-directed mutagenesis kit (Qiagen). Cy5-labeled primers JRH05.f/JRH05.r were used to PCR amplify the probes from the resulting plasmids, yielding the Cy5-labeled probes used in subsequent EMSAs.

The binding of GcsR to P_*gcvH2*_ was investigated by EMSA (58). A 200 nM sample of His_6_-GcsR was incubated with 2 nM Cy5-labeled P_*gcvH2*_ probe (specific probe) or 2 nM Cy5-labeled P_*PA5530*_ probe (nonspecific probe) in EMSA buffer (25 mM Tris-acetate [pH 8.0], 8 mM magnesium acetate, 10 mM potassium chloride, 1 mM dithiothreitol) for 30 min at 30°C. For EMSA reactions to examine the binding affinity of His_6_-GcsR for P_gcvH2_, 2.0 nM 5′-labeled Cy5 P_*gcvH2*_ probe was incubated with 0, 6.25, 12.5, 25, 50, 100, or 200 nM His_6_-GcsR. For EMSA reactions to determine the binding specificity of His_6_-GcsR for P_*gcvH2*_, a 200 nM sample of His_6_-GcsR was incubated with 2.0 nM 5′-labeled Cy5 P_*gcvH2*_ probe in the presence of 0, 25, 50, or 100 nM unlabeled P_*gcvH2*_ probe (specific competitor probe) or 100 nM unlabeled P_*PA5530*_ probe (nonspecific competitor probe). For EMSA reactions to determine the binding site of GcsR on the *gcvH2* promoter, a 200 nM sample of His_6_-GcsR was incubated with 2 nM each probe containing either a wild-type or a mutated binding site. For EMSA reactions to determine the binding of GcsR to the *mexE* promoter region, a 200 nM sample of His_6_-GcsR was incubated with 2 nM probe P_*mexE*_. The samples were then analyzed by PAGE under nondenaturing conditions and imaged with a Typhoon imager.

### Phosphatase assay.

Phosphatase activity of His_6_GcsR was measured as described previously for *E. coli* TyrR (25). Briefly, a 5 µM sample of His_6_-GcsR was incubated at 37°C for 3 h in phosphatase buffer (100 mM *p-*nitrophenylphosphate, 100 mM HEPES [pH 6.5]) in the presence of 2 mM glycine, 2 mM MgCl_2_, 2 mM ZnCl_2_, and 2 mM CoCl_2_ or in the absence of any divalent salts or glycine. Reactions were stopped by the addition of 1 ml of 1 M Na_2_CO_3_. The absorbance at 410 nm of the samples was measured with a Genesys 20 spectrophotometer to calculate the amount of substrate dephosphorylated.

### Spectroscopic measurement of extracellular pyocyanin

Experiments were done in triplicate. Cells were grown in 2 ml of PB at 37°C and 200 rpm for 24 h. Pyocyanin was measured as described previously (12). Briefly, cells were removed from the culture by centrifugation at 16,000 × *g* for 5 min and passage through Acrodisc syringe filters with 0.2-µm nylon membranes. The absorbance at 690 nm of the cell-free samples was measured with a Genesys 20 spectrophotometer and converted to the pyocyanin concentration by using a molar extinction coefficient of 4,130 M^−1^ cm^−1^.

### Paralytic-killing assay.

The *C. elegans* Bristol (N2) strain was cultivated under standard conditions (59). From synchronized populations of worms, hermaphroditic gravid adults were transferred to fresh nematode growth medium agar on a lawn of *E. coli* for 2 to 4 h to lay eggs and then removed. Eggs were incubated at 20°C for 3 days to the young adult stage (60).

Bacteria were grown overnight in BHI broth at 37°C with shaking. Cultures were diluted 1:100 in BHI broth, and 170-µl volumes were spread on BHI agar plates (1.7% Bacto agar, 60-mm petri plates) and then incubated at 37°C for 24 h (32). Synchronized worms were transferred to prepared BHI plates and incubated at room temperature for 6 h. Worms were examined for paralysis and scored for survival every 2 h with a stereomicroscope (×4.5 magnification). Worm paralysis was defined as no movement after mechanical stimulation, and death was defined as cessation of pharyngeal pumping. Data were analyzed with GraphPad Prism (GraphPad Software Inc.). Differences were considered significant when the *P* value was ≤0.05.

### Microarray data accession number.

RNA-Seq data were posted to the Gene Expression Omnibus (GEO) under accession number GSE76522.
