# Erratum for Sarwar et al., GcsR, a TyrR-Like Enhancer-Binding Protein, Regulates Expression of the Glycine Cleavage System in *Pseudomonas aeruginosa* PAO1

**DOI:** 10.1128/mSphere.00200-16

**Published:** 2016-07-27

**Authors:** Zaara Sarwar, Benjamin R. Lundgren, Michael T. Grassa, Michael X. Wang, Megan Gribble, Jennifer F. Moffat, Christopher T. Nomura

**Affiliations:** a Department of Chemistry, State University of New York—College of Environmental Science and Forestry, Syracuse, New York, USA; b Department of Microbiology and Immunology, State University of New York Upstate Medical University, Syracuse, New York, USA; c Center for Applied Microbiology, State University of New York—College of Environmental Science and Forestry, Syracuse, New York, USA; d Hubei Collaborative Innovation Center for Green Transformation of Bio-Resources, College of Life Sciences, Hubei University, Wuhan, People’s Republic of China

## ERRATUM

Volume 1, no. 2, doi:10.1128/mSphere.00020-16, 2016. On page 1, Importance section, line 5, “trichloroacetic acid cycle” should be “tricarboxylic acid cycle.” This error was due to a copyediting mistake.

